# Mapping the structural connections between the anterior cingulate cortex and the insula/ventrolateral prefrontal cortex

**DOI:** 10.1162/IMAG.a.1253

**Published:** 2026-05-26

**Authors:** Wei Tang, Javier Guaje, Shreyas Fadnavis, Sarah R. Heilbronner, Eleftherios Garyfallidis

**Affiliations:** Department of Psychological and Brain Sciences, Indiana University Bloomington, Bloomington, IN, United States; Program in Neuroscience, Indiana University Bloomington, Bloomington, IN, United States; Department of Intelligent Systems Engineering, Indiana University Bloomington, Bloomington, IN, United States; Bioscope AI, Boston, MA, United States; Department of Neurosurgery, Baylor College of Medicine, Houston, TX, United States

**Keywords:** anterior cingulate cortex, insula, ventrolateral prefrontal cortex, dMRI, cross-species neuroanatomy, salience network

## Abstract

The anterior cingulate cortex (ACC) is functionally closely related with the insula and the ventral lateral prefrontal cortex (vlPFC). The ACC and insula are the main hubs of the salience network, a set of functionally related brain regions involved in detecting salient stimuli and coordinating inter-network communication, while communication between ACC and vlPFC is closely linked to inhibitory control. However, despite strong indirect evidence from nonhuman primate (NHP) tract tracing, the structural connections between the ACC and the insula/vlPFC remain poorly understood in the human brain, in part because standard approaches fail to capture this projection. In this study, we show that diffusion-weighted magnetic resonance imaging (dMRI) can reconstruct ACC–insula/vlPFC pathways after a technical adjustment to the fiber orientation distribution functions. First, using NHP dMRI as a diagnostic tool, we pinpointed an issue of fiber orientation biases in the deep white matter that uniquely impacts the streamlines connecting the ACC and the insula/vlPFC region. We introduced a reweighting approach based on individual variability to correct for the fiber orientation density function. Finally, we demonstrated that, by applying this fix, dMRI tractography can fully recover the ACC–insula/vlPFC pathways, supporting the anatomical basis of the functional relationships among these regions.

## 1 Introduction

The neural circuitry connecting the anterior cingulate cortex (ACC) with the anterior insula and ventrolateral prefrontal cortex (vlPFC) plays an important role in cognitive and motivational control. Much of this insight comes from co-activation patterns in cognitive tasks (e.g., Daw et al., 2006; Hunt et al., 2018; Luk & Wallis, 2013; Rushworth et al., 2012), as well as functional connectivity among these regions during the resting state (Deen et al., 2011; Menon & Uddin, 2010; Taylor et al., 2009). Moreover, breakdown of such functional connectivity has been linked with psychiatric disorders (Szekely et al., 2017; Uddin, 2014; Whitton et al., 2019). These observations have led to the *salience network* theory, which posits that the ACC closely interacts with the insula (specifically, the anterior insula) to detect salient stimuli and switch between exogenous and self-related information processing (Menon, 2015; Menon & Uddin, 2010; Seeley, 2019). The vlPFC may also be an important part of this network (Trambaiolli et al., 2022). Furthermore, the ACC and vlPFC are important parts of the inhibitory control network (Apšvalka et al., 2022; Friedman & Robbins, 2022; Lee et al., 2014), with the vlPFC necessary for suppression of prepotent responses and the ACC responsible for situational monitoring for the purposes of implementing control. These networks are backed up by neuroanatomical studies in nonhuman animals. Tract-tracing studies in nonhuman primates (NHPs) have long established strong bidirectional projections among the three regions (Carmichael & Price, 1996; Schmahmann & Pandya, 2006; Trambaiolli et al., 2022; Vogt & Pandya, 1987). More specifically, Schmahmann and Pandya (2006) detailed the pathways of the ACC that traverse laterally through the deep white matter (WM) in the frontal lobe to reach the vlPFC and through the extreme capsule (EmC) to reach the insula. These fiber bundles are replicable by NHP diffusion-weighted magnetic resonance imaging (dMRI) tractography *in vivo*: streamlines derived from ACC and vlPFC seeds have shown mutual connections with each other (Neubert et al., 2015), and the dMRI-derived EmC has shown terminations in the ACC (Mars et al., 2016), both consistent with the tract-tracing work.

Despite findings from the NHP work, dMRI-derived ACC–insula/vlPFC pathways cannot be replicated in humans. Cross-species studies have pointed out that technical issues can cause tractography to deviate from anatomical ground truth (Grier et al., 2020; Maffei et al., 2021). It is thus a critical question whether the disparate finding from human studies is a result of technical issues or a true cross-species difference. Essentially, if no direct WM pathways exist between the ACC and insula/vlPFC in humans, a third region (or set of regions) may be mediating the many observed functional interactions (Cloutman et al., 2012), requiring modifications to current network theory.

We would argue that to conclude that the human brain contains no direct connection between the ACC and insula/vlPFC is premature. In fact, there is a biological basis for possible tractography failure in the ACC-related pathways. The deep WM region that contains the ACC–insula and ACC–vlPFC connections is occupied by thick anterior-posterior (A-P) running bundles. These bundles take up a substantial portion of the WM volume and dominate its dMRI signals. However, the bulk of the ACC–insula/vlPFC axons are oriented mostly medial-laterally. Thus, we hypothesized that fiber orientation models could assign disproportionally high probability toward the A-P direction and low probability toward the medial-lateral direction, causing erroneously low sampling rate and failure to catch ACC–insula/vlPFC fibers. This problem may be more pronounced in human brains, because the human frontal WM is much enlarged compared with NHPs’. Therefore, in this study, we examine the A-P bias of fiber orientation distribution functions (fODFs) and how it affects the identification of ACC–insula/vlPFC pathways. We develop a correction for this bias in the human by reweighting the fODFs based on cross-subject variability and are thus able to illuminate the “missing” ACC–insula/vlPFC pathways. Our results provide an anatomical basis for the functional networks linking the three regions.

## 2 Methods

### 2.1 Participants and procedure

T1-weighted images and dMRI data analyzed in this study were freely obtained from the Human Connectome Project (HCP) via ConnectomeDB (https://db.humanconnectome.org) and Primate Data Exchange (PRIME-DE) (https://fcon_1000.projects.nitrc.org/indi/PRIME/oxford2.html). The details of participants, procedure, and MRI acquisition have been documented in corresponding publications (Folloni et al., 2019; Glasser et al., 2013; Milham et al., 2018). All HCP subject recruitment procedures and informed consent forms, including consent to share de-identified data, were approved by the Washington University Institutional Review Board (Glasser et al., 2016). The ex vivo non-human primate samples used in this work were made available from an existing post-mortem collection at the University of Oxford. Because the work did neither impact nor involve decisions on the health, life, or death of any animal, additional ethical approval was not required (Folloni et al., 2019).

For the human data, a total of 174 individuals (age 22–36 years, 107 females) who had completed diffusion and structural scans were included from the HCP 7 T data pool. No participants were excluded. The NHP dMRI data contained post-mortem scans of six macaque monkeys (*Macaca Mulatta*, age 4.03–15.81 years, two females) with brains fixed in formalin via perfusion. Approximately 1 week before MRI scanning, the brains were immersed in phosphate buffer solution to enhance their diffusion signal. During scanning, the brains were suspended in Fomblin.

### 2.2 MRI acquisition and preprocessing

Details of the human 7 T diffusion and T1w image acquisition protocols are provided in the HCP reference manual (https://humanconnectome.org/study/hcp-young-adult/document/1200-subjects-data-release). Briefly, the dMRI acquisition used a spin-echo multiband (factor=2) protocol (TE = 71.2 ms; TR = 7 s; matrix size = 200 × 200; resolution = 1.05 mm isotropic voxels; 132 transversal slices). The acquisition used two gradient tables, each acquired once with anterior-to-posterior and posterior-to-anterior phase encoding polarities, respectively. Each gradient table included 65 unique diffusion weighting directions, two shells with b-values = 1000, 2000 s/mm^2^, and 6 b0 images. The full dMRI session lasted about 40 min. The preprocessing was performed as described by Glasser et al. (2013), based on the updated diffusion pipeline (v3.19.0), including distortion correction, eddy current correction, motion correction, gradient nonlinearity correction, and registration of the mean b0 image to native T1w images with FLIRT BBR+bbregister and transformation of dMRI.

The NHP dMRI acquisition and preprocessing pertaining to the public dataset are documented on PRIME-DE and in Folloni et al. (2019). Briefly, diffusion-weighted imaging was done on a 7T superconductive magnet driven by an Agilent DirectDrive console (Agilent Technologies, Santa Clara, CA, USA) using a 72 mm ID quadrature birdcage RF coil (Rapid Biomedical GmbH, Rimpar, Germany). Diffusion-weighted images were acquired by a spin-echo single line readout protocol (DW-SEMS: TE = 25 ms; TR = 10 s; matrix size = 128 × 128; resolution = 0.6 × 0.6 mm; 128 axial slices; slice thickness = 0.6 mm). Each subject’s dataset consisted of 16 non-diffusion-weighted (b = 0 s/mm^2^) and 128 diffusion-weighted (b = 4000 s/mm^2^) volumes with diffusion directions homogeneously distributed over the sphere. For preprocessing, eddy current correction was applied using the FDT tool by FSL.

### 2.3 Region masks for tractography and streamline evaluation

To evaluate the relative strength of ACC–vlPFC/insula connections in comparison with other frontal areas, in each species, a whole-brain tractogram seeding in the entire WM was summarized into between-region connectivity maps using regional masks. In the human, the region of interest (ROI) masks were created using the Desikan–Killiany atlas provided in FreeSurfer. First, surface labels of each parcel in the Desikan–Killiany atlas were converted to volumetric masks using the FreeSurfer mri_annot2vol command. The parcel masks were then combined into ROI masks following conventionally named cortical divisions. For example, two parcels “medialorbitofronal” and “frontalpole” in the Desikan–Killiany atlas were located in the ventromedial prefrontal cortex (vmPFC), and thus, merged to create the vmPFC mask. Table 1 lists the ROI masks and their corresponding parcels. The default ACC parcels were kept separate as ROIs for the dorsal and rostral (perigenual) ACC, so that if the tractography problem with the ACC–vlPFC/insula pathways was spatially specific (e.g., it affects one ACC subregion but not the other), the analysis could detect it.

**Table 1.** ROI masks for whole-brain tractography.

ROI name	Parcel names of the Desikan–Killiany atlas	Mask names of the CHARM4 atlas
vmPFC	Medialorbitofrontal, frontalpole	area_25, area_14
dmPFC	Superiorfrontal	area_8B, SMA/pre_SMA
dlPFC	Rostralmiddlefrontal, caudalmiddlefrontal	rostral_dlPFC, PM
OFC	Lateralorbitofrontal	areas_11/13, OFa-p, OLF, area_12m/o, cl_OFC,
vlPFC	Parsopercularis, parstriangularis, parsorbitalis	rostral_vlPFC, caudal_vlPFC, PMv*
insula	Insula	Ins/Pi
dACC	Caudalanteriorcingulate	area_24
rACC	Rostralanteriorcingulate	area_32

* From the CHARM5 atlas.

Abbreviations: vmPFC, ventromedial prefrontal cortex; dmPFC, dorsomedial prefrontal cortex; dlPFC, dorsolateral prefrontal cortex; OFC, orbitofrontal cortex; vlPFC, ventrolateral PFC; dACC, dorsal anterior cingulate cortex; rACC, rostral anterior cingulate cortex; SMA, supplementary motor area; PM, premotor; OFa, agranular orbitofrontal cortex; Ins, insula; Pi, parainsula.

Correspondingly, in the NHP, the ROI masks were created using the NMT v2.0 CHARM4 atlas (Jung et al., 2021) by merging the original volumetric masks in the atlas into conventionally named divisions of the prefrontal cortex (PFC). Minor modifications were applied to the original masks to make the NHP PFC divisions spatially align with those in the human: (1) The SMA/pre_SMA mask was extended posteriorly so that the ratio of area between the dorsomedial PFC (dmPFC) and the dACC was similar to that in the human, (2) the PM mask was merged into the dorsolateral PFC (dlPFC) so that the relative area of the dlPFC was similar to that in the human, and (3) The PMv mask from the CHARM5 atlas was merged into the vlPFC so that the spatial coverage of the vlPFC was similar to that in the human. The original masks and their corresponding merged divisions are listed in Table 1.

To compare the ACC–vlPFC/insula fiber trajectories across species, seed-based tractography was used. The seed ROI location was chosen to facilitate comparisons with the NHP tract-tracing data. Tract-tracing literature (Schmahmann & Pandya, 2006, Case 30) illustrates fiber trajectories between the ACC and vlPFC/insula via an injection into the region under the anterior-most end of the cingulate sulcus. Thus, the anterior-most end of the cingulate sulcus was used as a landmark to define the seed ROI in each species. To create the seed mask in the human, the anterior-most point of the cingulate sulcus was identified manually in FreeSurfer’s fsaverage template. Next, on the WM surface of the template brain, a parcel centered on this point was manually drawn with a diameter of 5 mm and saved as a FreeSurfer label. This label was then registered to each subject’s native brain surface using the package MNE-Python (v1.4.2)’s morph_labels function. Finally, in each subject, the label was converted to a volumetric mask in the dMRI space, using FreeSurfer’s mri_label2vol command, which was used as the ACC seed. To create the seed mask in the NHP, the anterior-most point of the cingulate sulcus was identified manually in the NMT v2.0 template (Jung et al., 2021). A cube centered on this point with five voxels in each dimension (approximately 5 mm in diameter) was hand drawn and saved as a volumetric mask. This mask was registered to each of the six animal’s dMRI space. In the native dMRI space, the cube mask was trimmed down to the portion intersecting with the cingulate cortex. Finally, the gray matter (GM)-WM boundary was identified by growing the trimmed mask into the WM by two voxels and keeping the WM voxels only. The remaining voxels were used as the ACC seed in subsequent tractography analysis.

To assess the number of streamlines terminating in the vlPFC/insula, two WM ROI masks were created, first in the template brain (NMT v2.0 template for the NHP; MNI152 1 mm T1 template (Fonov et al., 2009) for the human) and then registered to each subject/animal’s native space. Cross-species homology was a priority in determining the mask locations. For both species, the first mask (ROI 1) covered the WM region adjacent to the vlPFC, and the second mask (ROI 2) encompassed a segment of the EmC adjacent to the insula. Both masks were defined by WM voxels that fill the space confined by six anatomical landmarks demarcating the anterior, posterior, superior, inferior, medial, and lateral boundaries, as specified in Table 2. To locate these voxels, for each ROI, a 3D bounding box was manually drawn on the T1-weighted image of the template brain, such that the box minimally contained the six landmarks. A threshold was then applied to the values within the box, 500 for the NHP and 7000 for the human T1-weighted image. Voxels with an above-threshold value marked the WM within the box. To ensure there were no “holes” in this WM mask, 3D Gaussian smoothing was applied across the above-threshold voxels. The resulting binary volume was then used as the mask for the corresponding ROI.

**Table 2.** Landmarks defining the two WM ROIs.

	ROI 1	ROI 2
Anterior	The most anterior coronal slice of area 24	The most anterior coronal slice of the insula
Posterior	The most anterior coronal slice of the striatum	The most anterior coronal slice of the amygdala
Superior	The most superior axial slice of the principal sulcus (NHP)/inferior frontal sulcus (human)	The most superior axial slice of the insula
Inferior	The GM/WM boundary	The most inferior axial slice of the insula
Medial	The most medial sagittal slice of area 13	The striatal GM/WM boundary
Lateral	The GM/WM boundary	The insular GM/WM boundary

For the mean diffusivity and bundle profile analyses (Section 2.6), a third WM mask, ROI 3, was created in each species in the corresponding T1-weighted MRI space of the template used (ONPRC18 (Weiss et al., 2021) and XTRACT (Warrington et al., 2020) templates for the NHP, HCP1065 template (Yeh, 2022) for the human; see Section 2.6 for template details). The masks were manually drawn in FSLeyes following these steps for both species: (1) locate the coronal slice at the most anterior point of the cingulate sulcus, (2) in this slice, find the horizontal line from the most superior point of the orbitofrontal cortex to the cingulate surface, (3) using this line as the bottom side, draw and fill an in-plane square, and (4) expand the square in the A-P direction into a cube with equal expansion on each side. The WM region masked by the cube defines WM ROI 3.

### 2.4 Fiber orientation modeling and tractography

For both the human and NHP data, the same fiber orientation model and tractography methods were applied. All the analyses were implemented using DIPY (Garyfallidis et al., 2014). To estimate the fODF, a constant solid angle (CSA) model (Aganj et al., 2010) was fitted to each voxel with a spherical harmonic order of 6, resulting in a density function of 362 directions. Anatomically constrained tractography (ACT) (Smith et al., 2012) was applied, which uses tissue priors for the GM, WM, and cerebrospinal fluid (CSF) to determine the streamline trajectories and termination points during tracking. The GM/WM/CSF prior in each voxel was estimated using a Bayesian classification algorithm based on Markov random fields (Zhang et al., 2002). A probabilistic method based on particle filtering (Girard et al., 2014) was used to generate streamlines from the seed masks to the whole brain. In each voxel, six streamlines were generated from random seeding locations and tracked until they reached the GM in either the cortex or subcortical structures. Streamlines were discarded if they entered CSF or showed an excessive curving angle (< 30º) in the WM. To facilitate visualization, the 3D streamlines in each individual brain in all the figures were generated using deterministic tracking (Garyfallidis et al., 2014). While deterministic tracking was used for visualization, all the streamline statistics were calculated based on the probabilistic tracking results. These two approaches are complementary for solving the particular tractography problem in this study, and the rationale of this choice is further detailed in Section 4.

### 2.5 Streamline density in the cortical and WM ROIs and statistical tests

Streamline density in each voxel was calculated as the number of streamlines passing that voxel divided by the total number of valid streamlines generated. For visualization on the FreeSurfer-generated cortical surfaces, in each subject, the density scores between the pial surface and up to 2 mm into the WM were projected to the surface space using the FreeSurfer mri_vol2surf utility. Individual cortical maps were morphed to the fsaverage space for computing and visualizing the group average map.

To compare streamline density and labeled fiber patterns from tract-tracing literature, tract-tracing data were manually registered into the NHP dMRI space following these steps: (1) Images of the coronal sections in Schmahmann & Pandya (2006), Case 30, were imported into Adobe Illustrator (version 30.0, Adobe Inc., San Jose, CA, USA). (2) Matching coronal sections in the FA volume of one animal (sub-032319) were identified and the images were imported into Illustrator. (3) Digital contours of the GM, the EmC, the cord connecting the tracer injection site and the EmC were created by manually tracing the imported images in Illustrator. (4) For each matching pair of coronal sections, the digital contours were manually registered to the FA images by applying free-form transformations that maximize the alignment between cortical gray matter. (5) To register the major fiber bundles that occupy the deep WM between the ACC and lateral PFC, the image of coronal section 41 in Schmahmann & Pandya (2006), Page 536, was imported into Illustrator. Digital contours were created and manually registered to the FA images following the same steps 3 and 4.

For the whole-brain tractogram analysis, to compare streamline density across cortical regions (Table 1), a one-way ANOVA was conducted for each group comparison, with ROI as the factor, followed by post hoc Tukey’s HSD test between ROI pairs. For the seed-based tractogram analysis, to compare streamline density in WM ROIs 1 & 2 across species or before/after fODF reweighting (Section 2.8), an independent-samples *t* test was conducted for each comparison. All the *p* values were adjusted for multiple comparisons using Bonferroni’s correction.

### 2.6 Diffusivity directions and bundle profiles in WM ROI 3

First, we were interested in the diffusivity profile present in ROI 3. RGB color-coded vectors of the greatest diffusivity in ROI 3 were extracted from a dMRI reconstructed fiber template: the HCP-1065 young adult fiber template for the human (Yeh, 2022), and the ONPRC18 template for the NHP (Weiss et al., 2021). Each template provides the greatest diffusivity vector (V1) in each voxel in three directions, coded as RGB color channels, corresponding to the medial-lateral (M-L), the A-P, and the superior-inferior (S-I) directions, respectively. To compute the fraction of V1 in each direction, the fraction of RGB values was averaged across voxels.

Next, we were interested in which major fiber bundles occupy ROI 3, and so we used the XTRACT templates (Warrington et al., 2020). For each species, the un-thresholded bundle masks that overlap with ROI 3 were extracted. To measure the fraction of volume of each bundle within ROI 3, the number of voxels that overlap with ROI 3 was calculated for each bundle and divided by the total number of voxels across bundles.

### 2.7 Fiber orientation angle analysis

**Fig. 1.**
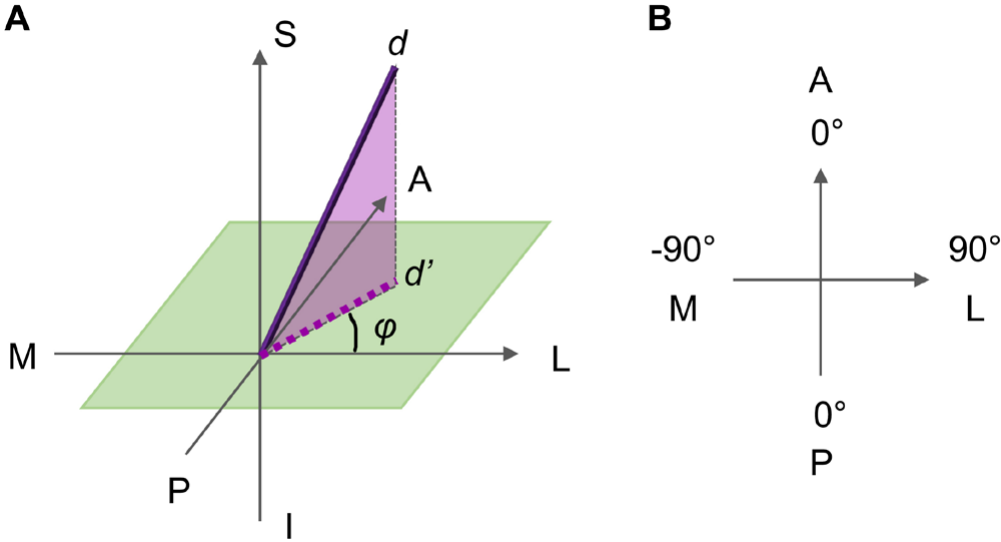
Definition of the fiber orientation angle. (A) Three-dimensional view of the orientation vector d and its associated angle ϕ. (B) In-plane view of ϕ and its value range.

The fODF is a function of orientation density on spherical coordinates. The coordinates determine the orientation vector *d* (Fig. 1A), and the density value corresponds to the probability of fibers in the corresponding orientation. There is an fODF for each voxel. To compare the estimated probability of A-P–oriented and medial-laterally oriented fibers in a chosen voxel, we define the orientation angle ϕ as follows: For a vector d in a given fODF, define its projection in the axial plane (spanned by the A-P and M-L axes) as d’. Angle ϕ is the angle between d’ and the M-L axis (Fig. 1A). Its value range is defined as follows (Fig. 1B): ϕ equals 0 when d’ completely aligns with the A-P axis regardless whether d’ points to the A- or P-direction; ϕ takes a negative sign and decreases from 0 to -90º as d’ move toward the right half of the M-L axis; ϕ equals -90º when d’ completely aligns with the right half of the M-L axis. Likewise, ϕ takes a positive sign and increases from 0 to 90º when d’ moves toward the left half of the M-L axis; ϕ equals 90º when d’ completely aligns with the left half of the M-L axis. Because the value is symmetric for the A- and P-half of the A-P axis, ϕ ranges in the interval [-90º, 90º].

### 2.8 Cross-subject variance reweighting

For a given fODF, the density of a particular orientation corresponds to the magnitude of vector d in that orientation. We computed the cross-subject standard deviation of the orientation density for each vector d in ROI 1. Then, in each subject, the density associated with each d was divided by its corresponding cross-subject standard deviation.

### 2.9 Orientation angle histogram

To assess how the SNR-based reweighting affects fODFs in different directions, histograms of ϕ were computed as follows: (1) In each subject, for each voxel in ROI 1, the probability values of the original fODF were subtracted from the probability values of the reweighted fODF in each direction. (2) The fODF differences were categorized into increase (positive difference) and decrease (negative difference). (3) In each subject, separately for the increased and decreased fODFs, the difference values were pooled over the 362 directions and over all the voxels in ROI 1. The median of each pooled population was calculated as threshold. (4) In each subject, for increased fODFs, the d vectors associated with above-median differences were identified. A histogram of ϕ angles associated with these vectors was computed over 20 equal-sized bins spanning the [-90º, 90º] interval. For decreased fODFs, the histogram was calculated in the same manner but for d vectors associated with below-median values. (5) The histograms were then averaged across subjects.

## 3 Results

### 3.1 Absence of the medial-laterally oriented ACC pathways in human dMRI tractography

**Fig. 2.**
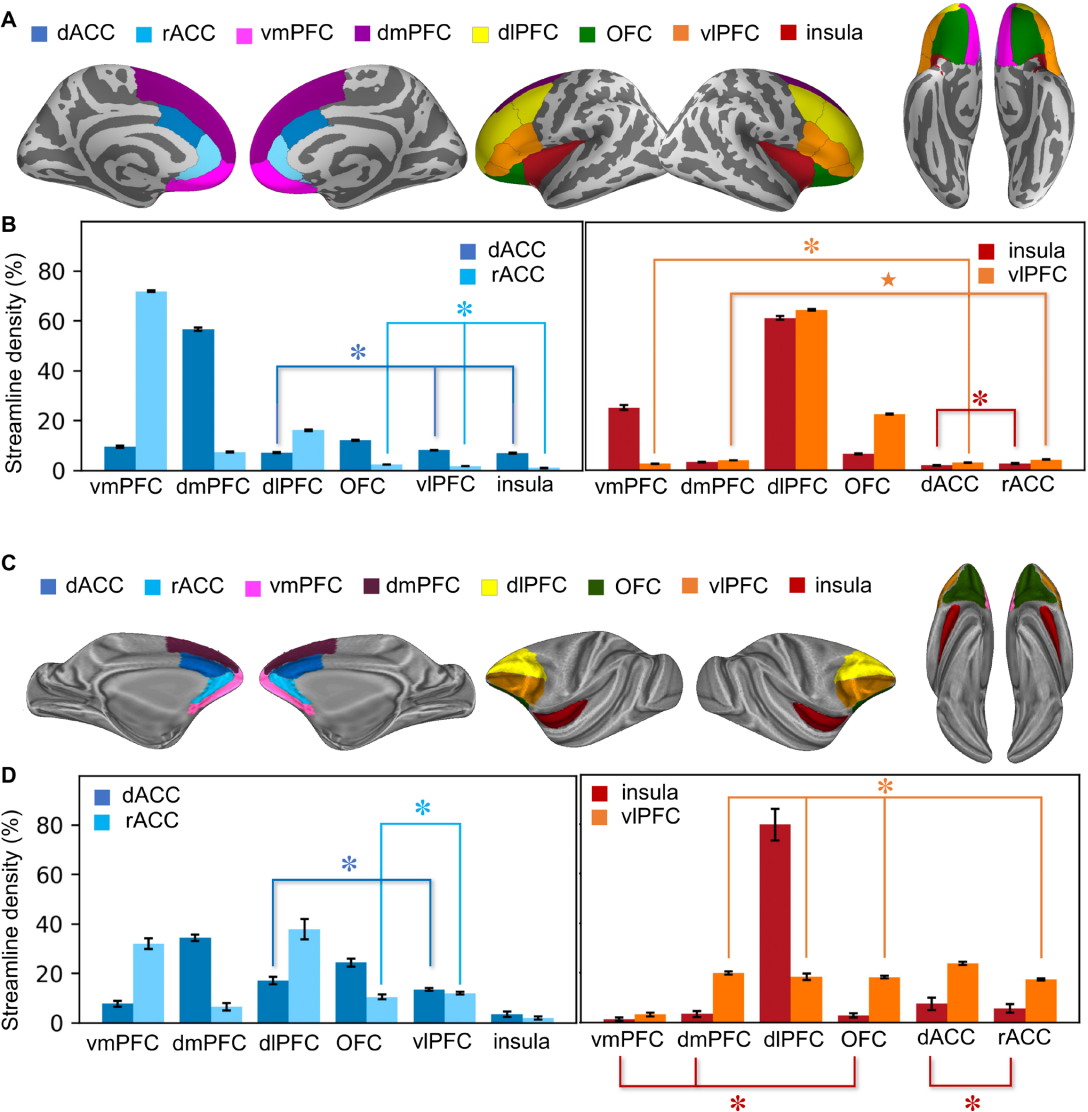
Streamline density among frontal regions. (A) ROIs used for the analysis in the human, shown on the inflated surface of the fsaverage brain template. (B) Streamline density (normalized over all target ROIs) from each seed region in the human. Symbols above the bars indicate Tukey’s HSD test outcome, such that groups sharing the same symbol show no significant difference from each other but are significantly different from all the other groups. (C) ROIs used for the analysis in the NHP, shown on the inflated surface of the NMT v2.0 brain template. (D) Streamline density (normalized over all target ROIs) from each seed region in the NHP. Symbols follow the same scheme as in (B).

We evaluated the relative strength of ACC–vlPFC/insula connections in comparison with other frontal areas, first in the human (Fig. 2A & 2B) and then in the NHP (Fig. 2C & 2D). From a whole-brain tractogram, we calculated streamline densities connecting the dACC and rACC to the insula and vlPFC, as well as between these four regions and different ROIs within the PFC. In the human, streamlines from the dACC and rACC terminating in the insula and vlPFC were less dense compared with streamlines connecting dACC and rACC to other regions (Fig. 2B, one-way ANOVA main effect of target region, dACC seed: *F*_1, 5_ = 22009.23, *p* < 1×10^-10^; rACC seed: *F*_1, 5_ = 2964.42, *p* < 1×10^-10^). Post hoc Tukey’s HSD tests showed that for both dACC and rACC seeds, streamline densities within the insula and vlPFC were indistinguishable from each other, but they were significantly lower than streamline densities within the vmPFC and dmPFC. For the dACC seed, streamline density in the dlPFC was roughly equivalent to those in the insula/vlPFC; for the rACC seed, similarly low density was found with the OFC. Together, these patterns suggest that streamlines from the dACC and rACC struggle to reach the insula and vlPFC. In addition, in general, they have more difficulties reaching the lateral relative to the medial surface of the PFC. Reciprocally, streamlines from the insula and vlPFC terminating in both ACC regions were less dense compared with streamlines connecting insula/vlPFC to other PFC ROIs (Fig. 2B, one-way ANOVA, insula seed: *F*_1, 5_ = 2059.56, *p* < 1×10^-10^; vlPFC seed: *F*_1, 5_ = 18855.28, *p* < 1×10^-10^). Post hoc Tukey’s HSD tests showed that for the insula seed, streamline densities within the dACC and rACC were indistinguishable from each other, but they were significantly lower than those connecting to the other target regions. For the vlPFC seed, streamline densities in the dACC and rACC were at the same low level as in the vmPFC and dmPFC. Together, these patterns suggest that streamlines from the insula and vlPFC have difficulties reaching the dACC and rACC. In addition, in general, they have more difficulties reaching the medial relative to the lateral surface of the PFC.

In the NHP, streamlines from the dACC and rACC terminating in the PFC regions showed more evenly distributed strength across regions than those in the human, while the rank-order of strength among regions was similar across species (Fig. 2D, one-way ANOVA main effect of target region, dACC seed: *F*_1, 5_ = 69.11, *p* < 1×10^-10^; rACC seed: *F*_1, 5_ = 41.02, *p* < 1×10^-10^). Likewise, streamlines from the insula and vlPFC terminating in both ACC regions showed more evenly distributed strength than those in the human (Fig. 2D, one-way ANOVA, insula seed: *F*_1, 5_ = 90.66, *p* < 1×10^-10^; vlPFC seed: *F*_1, 5_ = 72.29, *p* < 1×10^-10^). The rank-order of strength among regions differed across species, especially for the vlPFC seed, indicating possible impact of anatomical dishomology (e.g., the expansion of PFC in the human) on the tractography results.

### 3.2 Comparative analysis to evaluate the underlying cause of low connectivity

**Fig. 3.**
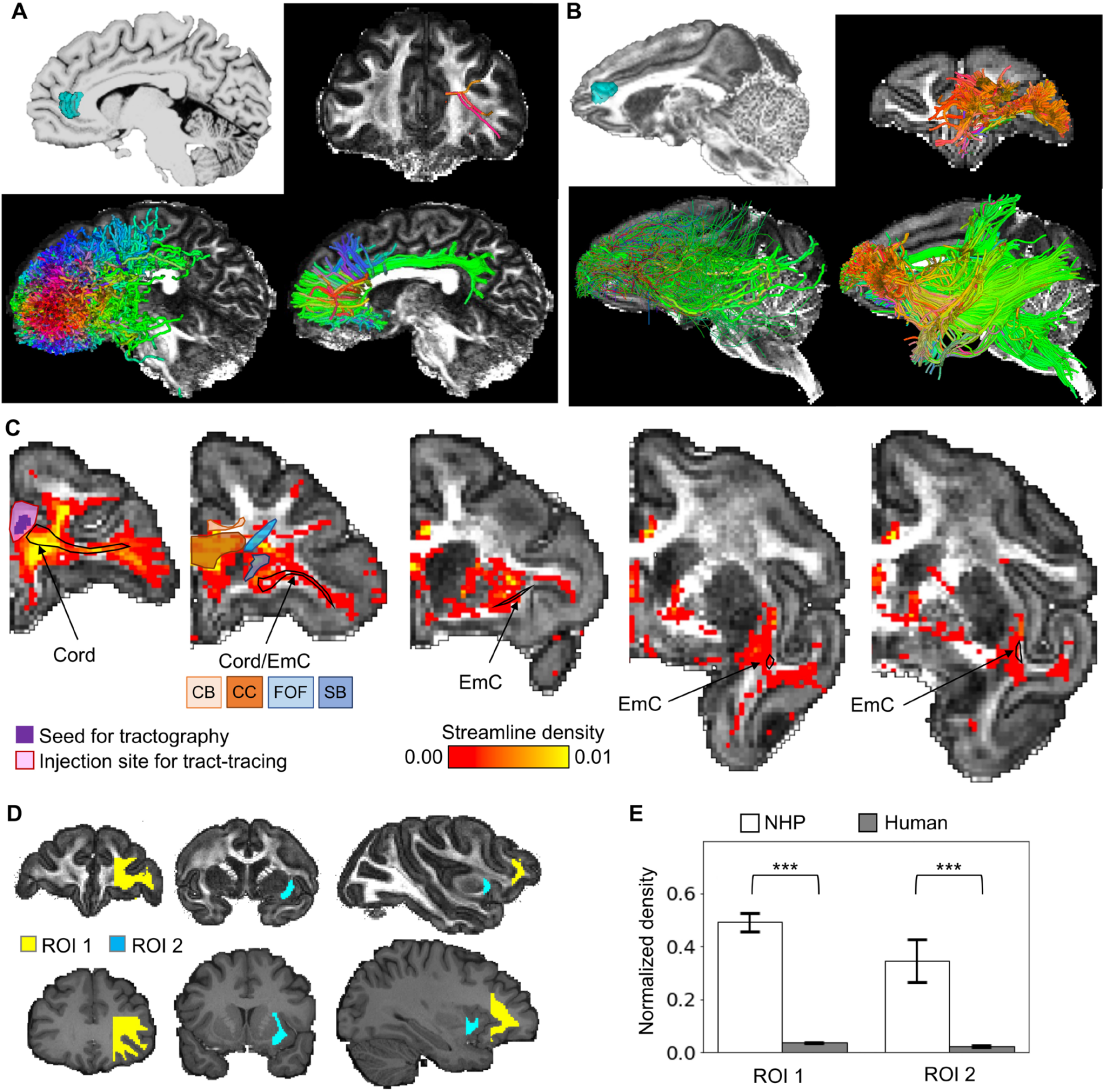
Absence of medial-lateral connections in human dMRI tractography. (A) Tractography result from a single HCP subject (100610), showing the ACC seed location (top left), the streamlines reaching the vlPFC in a coronal view (top right), all the probabilistic streamlines in a sagittal view (bottom left), and all the deterministic streamlines in a sagittal view (bottom right). (B) Tractography result from a single NHP subject (sub-032319), showing the ACC seed location (top left), the streamlines reaching the vlPFC in a coronal view (top right), all the probabilistic streamlines in a sagittal view (bottom left), and all the deterministic streamlines in a sagittal view (bottom right). For visibility of the ACC–insula connections, the probabilistic streamlines not entering the EmC were displayed with reduced thickness and brightness. (C) Probabilistic streamline density in the NHP displayed on coronal slices, indicating the course of the connections from the ACC seed to the vlPFC/insula through the EmC. Black contours mark the tract-tracing labeled fiber locations with an injection that matched the seed location. Colored contours on section #2 mark the locations of major fiber bundles in the deep WM between the ACC and the vlPFC, according to Case 30 in Schmahmann & Pandya (2006). Abbreviations: CB, cingulum bundle; CC, corpus callosum; FOF, fronto-occipital fasciculus (or IFOF by convention); SB, subcortical bundle. (D) Locations of ROIs 1 & 2 in the NHP and the human. (E) Normalized streamline density (log-transformed for statistical comparison) of ROIs 1 & 2 in the NHP and the human. ***p < 1× 10^-10^.

To evaluate the underlying cause of the scarce medial-lateral connections in the human, we analyzed the trajectories of a subset of the streamlines, those originating from an area near the anterior end of the cingulate sulcus (Fig. 3A). For visual inspection, we used deterministic tractography to better delineate and compare fiber trajectories, after verifying that the overall patterns of the probabilistic and deterministic streamlines were consistent (Fig. 3A, bottom panels). The seed ROI was chosen to facilitate comparisons with the NHP tract-tracing data (Schmahmann & Pandya, 2006, Case 30) that suggests that axonal fibers from this seed region travel through the EmC to reach the insula, following a medial-lateral trajectory in the frontal deep WM to reach the vlPFC. However, in the human brains, we observed an absence of the above-described pathways in the tractography results, illustrated in an example subject in Figure 3A. Indeed, in 122 of the 174 HCP subjects, the entire EmC was absent when seeding from the ACC (Supplementary Figures S2–S7). In contrast, tractography with NHP dMRI produced results consistent with the tract-tracing literature (Fig. 3B & 3C). The streamlines originating from a matching seed location traveled laterally to reach the vlPFC region in the same coronal plane as shown by Schmahmann & Pandya (2006) (Fig. 3B, upper right; Fig. 3C, first coronal section). Another group of streamlines first traveled laterally and then caudally to join the EmC and reach the insula (Fig. 3B, bottom panels, showing consistent results between probabilistic and deterministic tractography). To further validate streamline trajectories with tract-tracing data, we manually registered the tracing-identified contours of the EmC, and the cord that connects the ACC seed region and the EmC, into the NHP dMRI space (see Section 2.5) (Fig. 3C). Across coronal sections, the progression pattern of streamline density calculated from the result in Figure 2B overlapped substantially with that of the registered contours, supporting the validity of the NHP tractography results. These results stand in contrast to those from the human.

To test the statistical significance of the observed cross-species difference, we compared streamline densities between the human and NHP brains in two WM ROIs that contained the bulk of the tract-tracing suggested pathways (Fig. 3C). ROI 1 was situated between the cingulate gyrus and the lateral prefrontal cortex, and ROI 2 was situated between the putamen and the insular cortex. Their specific boundaries were determined by landmarks chosen to maximize cross-species homology (see Section 2). To control for baseline difference of streamline density across species, we divided the cross-voxel average density in each ROI by the cross-voxel average density in the entire WM in each species. In both ROIs, the normalized density in the monkey (averaged across 6 animals) was highly significantly greater than the normalized density in the human (averaged across 174 individuals) (independent-sample *t* tests, ROI 1: *t* = 36.70, *p* < 1× 10^-10^; ROI 2: *t* = 16.88, *p* < 1× 10^-10^).

**Fig. 4.**
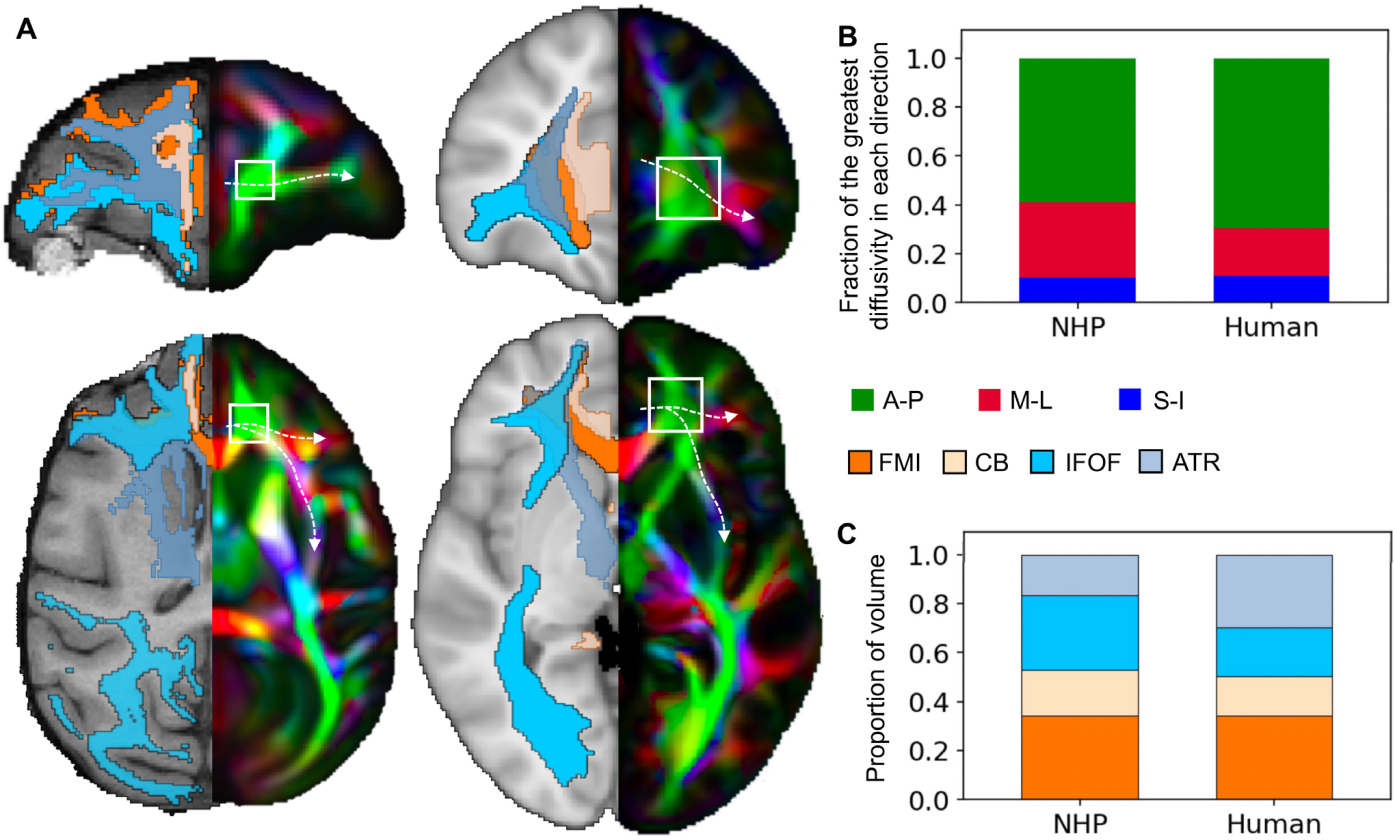
Comparative analysis of bundle profiles and diffusivity in the deep WM region between the ACC and lateral cortices. (A) Bundle profile (left hemisphere) and color-coded, FA-weighted mean diffusivity map (right hemisphere) in the NHP (left) and human (right), provided by dMRI templates in each species (see Section 2), overlayed on the T1-weighted images accompanying the template. White squares mark the boundaries of WM ROI 3. Arrows mark the suggested trajectories of fibers connecting the ACC and the vlPFC/insula. (B) Mean diffusivity in each direction, averaged across voxels in ROI 3. (C) The volumetric proportion of each bundle in ROI 3. Acronyms: A-P: anterior-posterior, M-L: medial-lateral, S-I: superior-inferior, FMI: forceps minor, CB: cingulum bundle, IFOF: inferior frontal-occipital fasciculus, ATR: anterior thalamic radiation.

We speculated that cross-species differences in the anatomy may explain the above observations. Such differences may lead to a general issue for tractography in the human, which is not tied to the specific datasets in this study. To further delineate these differences, we analyzed anatomical profiles of the deep WM region, ROI 3, in atlases in both species (see Section 2.6). First, in both the NHP and the human, a majority of voxels in ROI 3 have the highest diffusivity in the A-P direction (Fig. 4A), with the A-P dominance more extreme in the human (Fig. 4B). Second, in both the NHP and human, four major bundles occupy this region (Fig. 4A): the cingulum bundle (CB), the forceps minor (FMI), the anterior thalamic radiation (ATR), and the inferior frontal-occipital fasciculus (IFOF), in the order of medial to lateral positioning. The anterior part of the IFOF extends laterally into the vlPFC, while the middle part of it joins the EmC to reach the insula. In ROI 3, all of the four bundles travel anterior-posteriorly; fibers connecting the ACC with the vlPFC/insula must cross the CB, FMI, and ATR medial-laterally to join the IFOF. We have observed this trajectory with tractography in the NHP (Fig. 3B) but not in the human. Indeed, for the human, when we extended the ACC seed region to include both medial and lateral cortices, only the laterally originated streamlines could reach the IFOF (Supplementary Fig. S1). This was partly because the physical distance between the ACC and the vlPFC is bigger in the human (approximately twice of the distance in the NHP), which increases the difficulty of consistently tracking in a direction with weak diffusivity across voxels. Moreover, the relative sizes of the ATR and IFOF differ between species: compared with NHP, the human ATR was proportionally larger, whereas the IFOF was proportionally smaller (Fig. 4C). This could also increase the difficulty for streamlines to cross the ATR and reach the IFOF in the human.

### 3.3 A-P bias in the fODFs of the deep WM

**Fig. 5.**
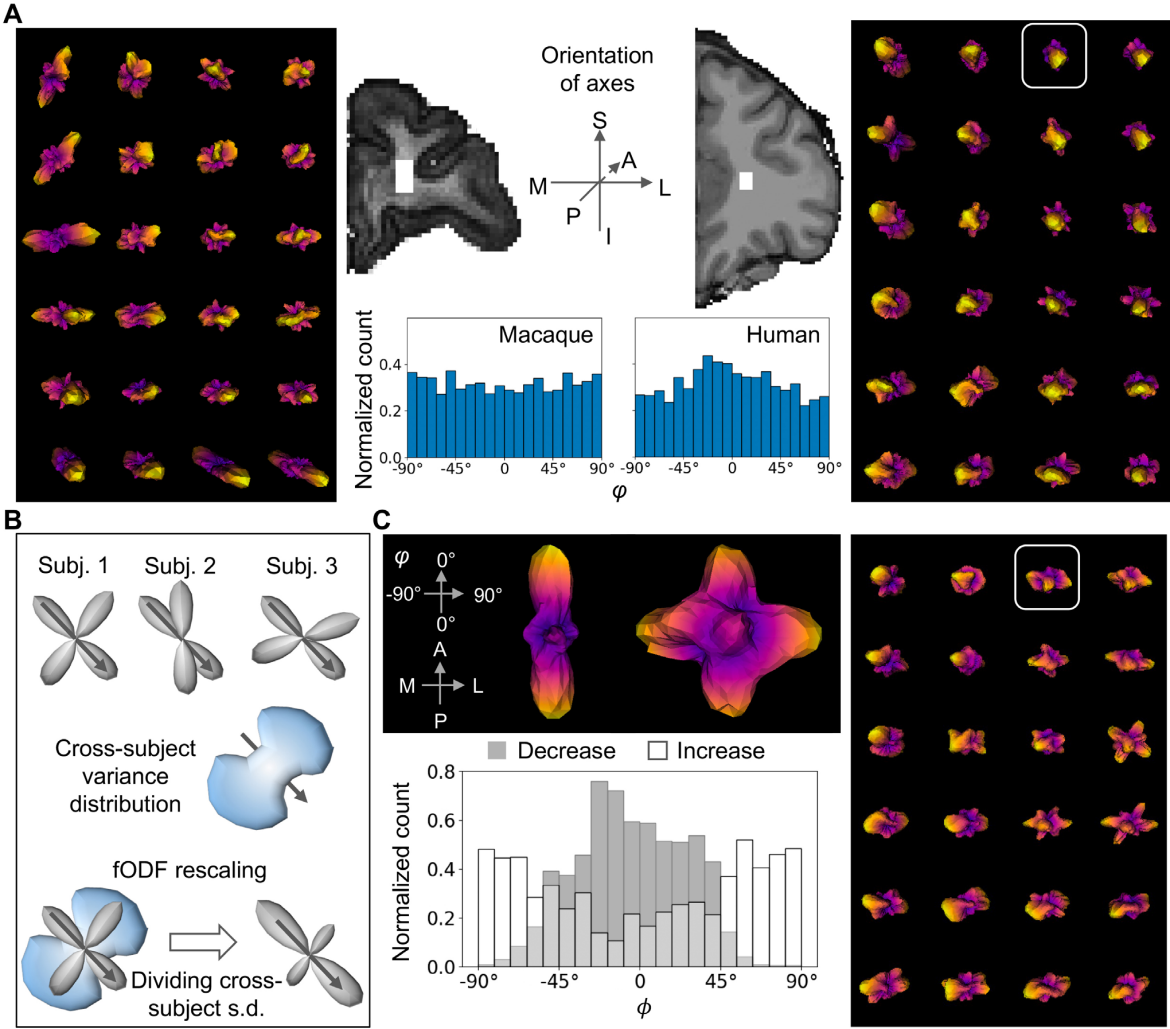
Reweighting fODFs to boost the probability in the directions of existing fibers. (A) Comparison of fODFs in the NHP and human brains. Middle panel shows two coronal slices from an NHP and a human brain, respectively, with the white masks marking the voxels for comparison. The fODFs of voxels from each mask are shown in the left and right panels, respectively. The axes of the 3D fODF are shown by the schematic in the middle panel. The histograms of angle are shown at the bottom. Abbreviations: A=anterior, P=posterior, S=superior, I=inferior, M=medial, L=lateral. (B) Schematics showing the rationale of SNR-based reweighting of fODFs. Top: Schematic example of the variance across three fODFs from three subjects (“Subj.”). Gray arrow marks the density resulting from the dMRI signal of a true fiber bundle running in the pointed direction, and this bundle consistently exists in each subject. Middle: Schematic variance function across the three fODFs. The variance is low in the arrow-pointed direction because presumably the true fiber bundle generates consistent dMRI signals across subjects. The variance in the other directions is high because presumably the density in these directions is resulted from random noise. Bottom: Schematic of the fODF reweighting. Density in the arrow direction proportionally increases after the fODF is divided by the cross-subject standard deviation. (C) Reweighting applied to the fODFs of the same example human subject in A. Reweighted fODFs from the same voxels are shown in the right panel. Middle top: the reweighting effect on a single fODF from the voxel highlighted in the right panels (left: before reweighting, right: after reweighting). The fODF is reoriented for visualizing the density in the M-L direction. Middle bottom: Histogram associated with increased and decreased probability due to reweighting.

Based on the observations above, to probe for a solution that can more accurately track medial-lateral fibers, we examined the fODFs in ROI 3. Visual inspection suggested that the fODFs in the human had a strong bias toward the A-P axis, in contrast to the more uniform fODFs in NHPs. To illustrate, we extracted fODFs from a coronal slice in a macaque and a human brain (Fig. 5A middle panel, MR images), with the voxel locations matched across species. Compared with the fODFs in the monkey (Fig. 5A left panel), fODFs in the human (right panel) showed high probability mass along the A-P axis and much lower probability mass along the M-L axis. To examine whether this difference was robust across individuals, for each subject, we computed the histogram of orientation angles (see Section 2.7 for the definition of ϕ) associated with above-median probability in the fODFs. The angles ϕ of voxels in ROI 3 were pooled for computing each histogram, and the histograms were averaged across subjects in each species. In NHPs, the orientation angles associated with above-median probability were evenly distributed over the interval [-90º, 90º], while in humans high counts tended to center around 0º (Fig. 5A middle panel, bottom plot). Thus, high probability in the A-P direction (ϕ = 0º) is more frequently observed in humans than in NHPs. The A-P bias in humans may help to explain why the ACC–insula/vlPFC pathways can be identified by dMRI tractography in NHPs but not in humans.

The fODF A-P bias may be a resolvable technical issue, rather than an underlying biological reality. It is possible that the disproportionate bundle sizes cause unbalanced signal-to-noise ratio (SNR) in the fODFs that impact tractography. The tracking algorithm might have sampled frequently in the A-P direction while failing to distinguish probability peaks generated by axonal diffusion signal or by noise in the M-L direction. In order to address this issue, we aimed to distinguish fODF peaks generated by axons from those generated by random water diffusivity. Our strategy was to reweight the fODF by a measure of cross-subject variability. Our logic was that if M-L axons are consistently present across subjects in the same region, then the fODF peaks in this region and direction would be consistent across subjects, while peaks driven by noise would randomly vary (Fig. 5B, top). Thus, low cross-subject variance would indicate that the dMRI signal in the corresponding direction is likely driven by existing axons (Fig. 5B, middle). Following this rationale, in each subject and for each voxel in ROI 3, we divided the fODF by the cross-subject standard deviation (Fig. 5B, bottom).

The reweighting procedure resulted in increased probability in the M-L axis of the fODFs (Fig. 5C). To illustrate this effect, we plotted an example fODF from a single voxel (Fig. 5C, middle panel). Before reweighting (left), the fODF peaks were highly aligned with the A-P axis; after reweighting (right), additional peaks were observed along the orthogonal M-L axis. Similar effects can be seen in most of the voxels shown in the right panel of Figure 5C: after reweighting, many fODF peaks became visible in the plane orthogonal to the A-P axis. To test whether this was a robust effect across voxels and subjects, we measured the histogram of orientation angles in which the fODF showed above-median increase or decrease after reweighting (see Section 2). The decrease was mostly found around 0º (A-P axis), while the increase was mostly found around ± 90º (R-L axis) (Fig. 5C, middle panel).

### 3.4 Tractography with reweighted fODFs

**Fig. 6.**
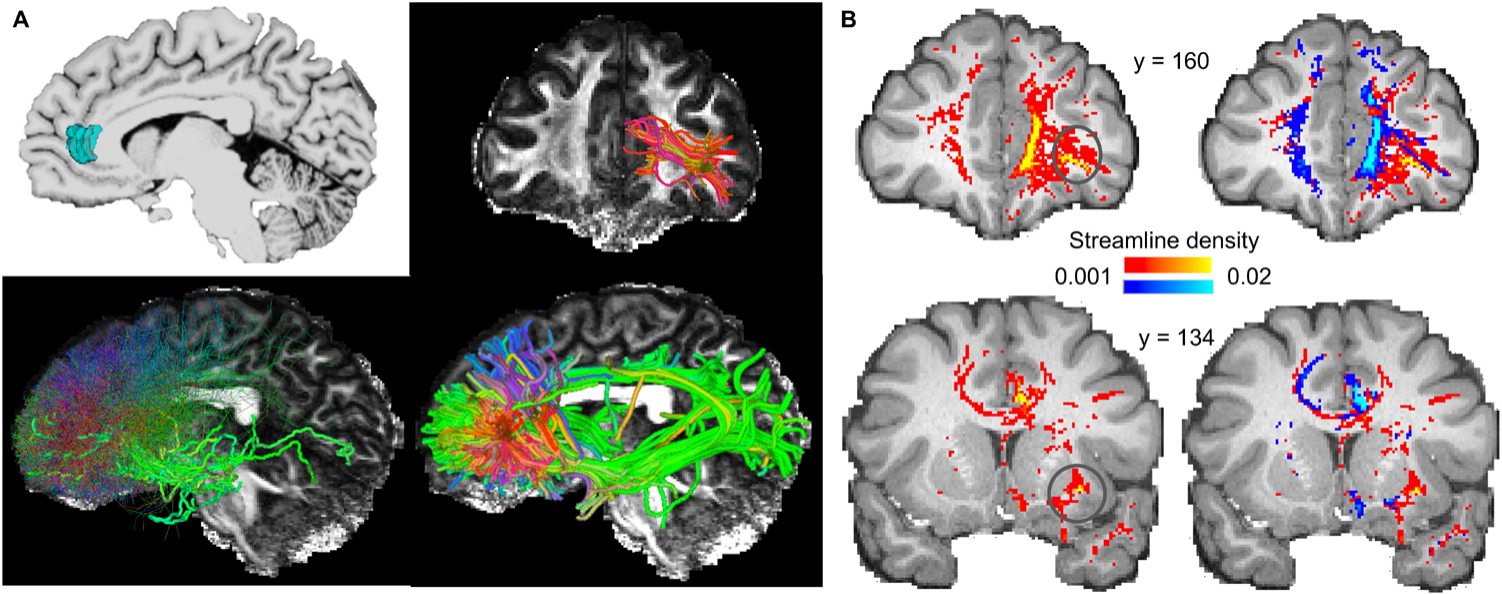
Recovery of the pathways after fODF reweighting. (A) Tractography result from the same HCP subject (100610), showing the ACC seed location (top left), the deterministic streamlines reaching the vlPFC in a coronal view (top right), all the probabilistic streamlines in a sagittal view (bottom left), and all the deterministic streamlines in a sagittal view (bottom right). For visibility of the ACC–insula connections, the probabilistic streamlines not entering the EmC were displayed with reduced thickness and brightness. (B) Streamline density of the same ACC seed, showcasing the density before and after reweighting. Left: two representative coronal MRI slices showing the density after reweighting, with the circles marking the regions of streamlines terminating in the vlPFC (top) and the insula (bottom). Right: the same MRI slices showing the density before reweighting (blue colormap), superimposed on the density after reweighting.

**Fig. 7.**
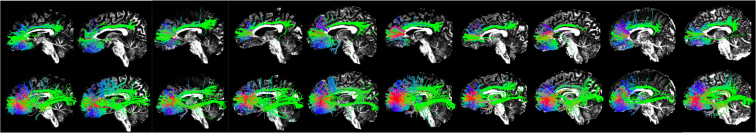
Tractograms before (top) and after (bottom) fODF reweighting in 10 HCP subjects.

**Fig. 8.**
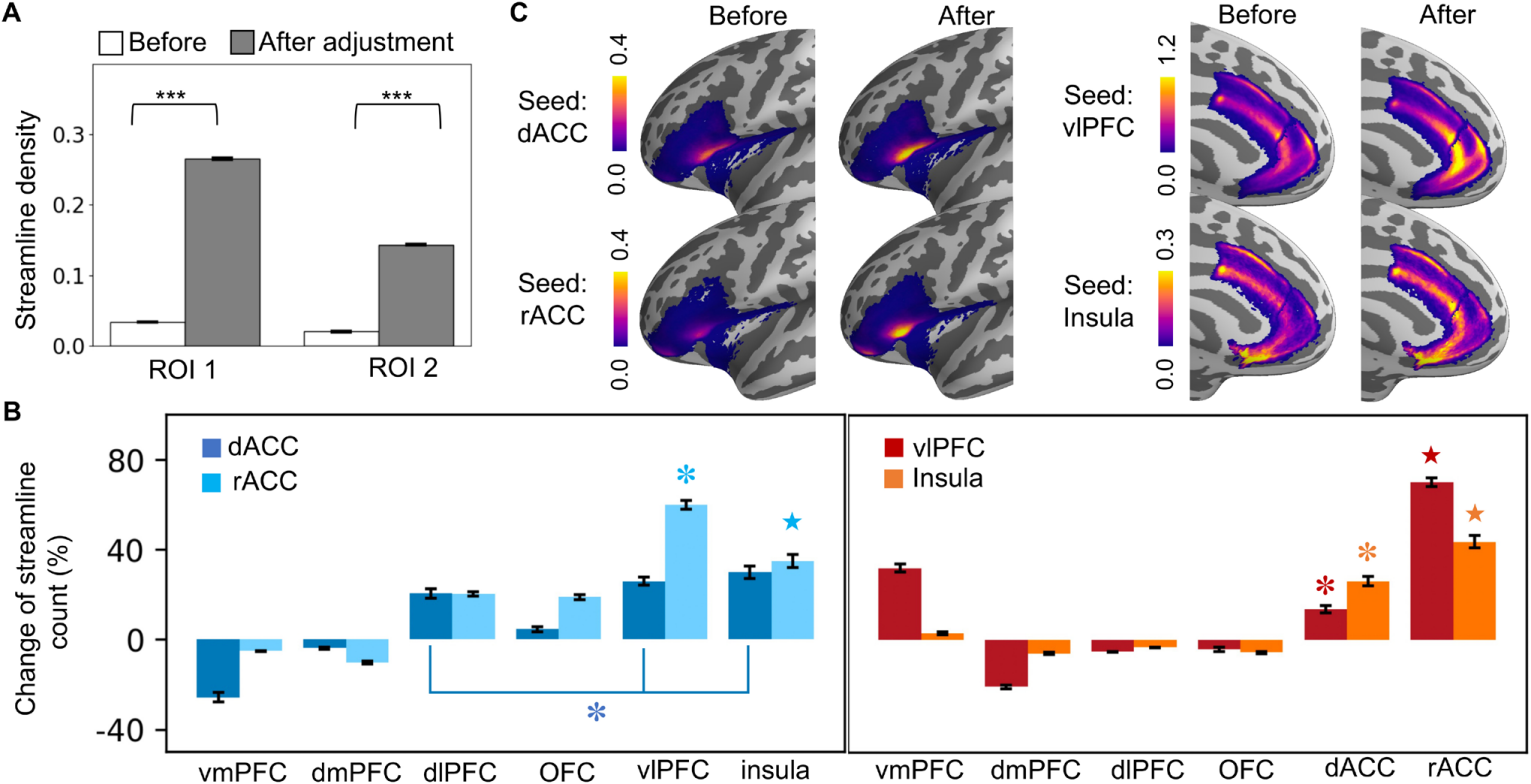
Cross-subject average of streamline density before and after fODF reweighting. (A) Mean streamline density (log-transformed) across subjects in ROIs 1 & 2. ***p < 1× 10^-10^. (B) Percentage change of streamline density after fODF reweighting compared with that before reweighting. Symbols above the bars indicate Tukey’s HSD test outcome, such that groups sharing the same symbol show no significant difference from each other but are significantly different from all the other groups. (C) Streamline density before and after fODF reweighting, shown in the insula & vlPFC (left panel) and the dACC & rACC (right panel) on the inflated surface of the fsaverage brain template.

After reweighting the fODFs of ROI 3 in each subject’s native space, we regenerated streamlines from the same ACC seed region (Fig. 6A). We first compared streamline trajectories before and after the reweighting. In the example HCP subject, visual inspection suggested that reweighting resulted in the recovery of both pathways connecting the ACC seed to the insula and vlPFC (Fig. 6A). We observed a full bundle oriented in the M-L direction connecting with the vlPFC (Fig. 6A, upper right). Moreover, the EmC was clearly identifiable (Fig. 6A, lower right). This change was consistent with the observed increase of streamline density in ROIs 1 & 2 (Fig. 6B). We then verified the robustness of this recovery effect across subjects. The increased M-L streamlines and recovered EmC were observed in 115 out of the 122 HCP subjects in which the EmC was absent before reweighting (Fig. 7 & Supplementary Figures S2–S7). There was also a significant increase of streamline density in ROIs 1 & 2 (Fig. 8A, paired-sample *t* test across subjects, ROI 1: *t*_173_ = -24.25, *p* < 1× 10^-10^; ROI 2: *t*_173_ = -17.61, *p* < 1× 10^-10^).

Importantly, the increase of streamlines was specific to the ACC–insula/vlPFC pathways rather than globally observed in the M-L PFC connections (Fig. 8B). We repeated the analyses in Figure 1B and compared streamline density before and after fODF reweighting. The reweighting resulted in a significant increase from both the dACC and rACC seeds to the insula and vlPFC targets (Fig. 8B, one-way ANOVA main effect of target region, dACC seed: *F*_1, 5_ = 127.04, *p* < 1×10^-10^; rACC seed: *F*_1, 5_ = 270.92, *p* < 1×10^-10^), as well as from the insula and vlPFC seeds to the dACC and rACC targets (Fig. 8B, one-way ANOVA main effect of target region, insula seed: *F*_1, 5_ = 189.19, *p* < 1×10^-10^; vlPFC seed: *F*_1, 5_ = 602.03, *p* < 1×10^-10^). Post hoc Tukey’s HSD tests showed that for the dACC and rACC seeds, the increase was the highest for the insula and vlPFC compared with the other target regions (except for the dlPFC with the dACC seed, which had a similar increase to that of the insula and vlPFC). For the insula and vlPFC seeds, the increase was the highest in the dACC and rACC compared with the other target regions (except for the dACC with the insula seed, which had less increase than that of the vmPFC). The detailed streamline density maps before and after reweighting in the dACC, rACC, insula, and vlPFC are shown in Figure 8C.

## 4 Discussion

Identifying pathways connected with the cingulate cortex has been a challenge for dMRI tractography. The cingulum bundle and other A-P–oriented large bundles (such as the superior longitudinal fasciculi) tend to cause predominantly high probability mass in the fODFs. As a result, streamlines connecting the medial wall with the lateral cortex are difficult to track and prone to false negative outcomes. This issue can lead to erroneous understanding of anatomical connections and related functional interpretations of the ACC and its networks. In this report, we show that the “missing” connections between the ACC and the insula and vlPFC are likely a false negative that can be corrected by reweighting fODFs based on individual variability. By consulting the NHP tract-tracing and tractography data, we were able to diagnose the underlying technical issues and come up with the solution accordingly. The corrected tractography recovered the ACC–insula/vlPFC pathways that support important functions but have been poorly understood in the human brain.

### 4.1 Anatomical connectivity underlying the salience network

Our findings open the door to exploring the anatomical basis of the salience network. The classical definition of the salience network (Menon & Uddin, 2010) highlights two core nodes, the dACC and the insula. While their functional relationships have been extensively discussed in the literature (Cushnie et al., 2023; Uddin, 2014), the anatomical connections to support their interactions remain unclear in humans (Cloutman et al., 2012). A potential cause of this problem is the difficulty tracking streamlines between the medial and lateral cortices as described above. Here, we show that the EmC, a bundle running longitudinally and adjacent to the insula, carries fibers from the ACC. Thus, the fODF reweighting in ROI 1 can be a common fix for tracking streamlines that connect different ACC subregions and the insula, including nodes of the salience network.

Another relevant finding is the connections between the ACC and the vlPFC. While the majority of salience network studies are focused on the ACC and insula, whether the vlPFC is also a node or not remains an unresolved question (Trambaiolli et al., 2022). In value-based decision making, the functions of the vlPFC and dACC are closely related in both humans and NHPs (Daw et al., 2006; Hunt et al., 2018; Luk & Wallis, 2013; Monosov & Rushworth, 2021). Whether the dACC and possibly the vlPFC in these studies are part of the salience network will bring fundamental insights to advance both fields. Moreover, subregions of the ACC are often found to co-activate with the vlPFC in tasks involving inhibitory control (Apšvalka et al., 2022; Friedman & Robbins, 2022; Lee et al., 2014). Whether such co-activation reflects direct interaction or the modulation by a third region will lead to different understanding of the underlying neural mechanism. These questions cannot be resolved with functional data alone because functional interactions can arise from indirect anatomical connections. In the NHP tract-tracing literature, strong bidirectional projections have been established among the ACC, vlPFC, and insula (Carmichael & Price, 1996; Schmahmann & Pandya, 2006; Trambaiolli et al., 2022; Vogt & Pandya, 1987). Here we show that some of these anatomical pathways can be replicated in humans using dMRI tractography. Importantly, we found both vlPFC and insula connections with the same ACC seed. The approach can be directly applied to the other ACC subregions to explore whether vlPFC and insula connections converge onto an ACC subregion that is part of the salience network.

### 4.2 Anterior origin of the EmC in the medial PFC

Previous work on dissecting the EmC with dMRI tractography has been focused on its temporal and occipital segments (e.g. Makris & Pandya, 2009; Mars et al., 2016; Radwan et al., 2022), and not much discussion was on terminations in the frontal cortex. Our results suggest that fibers from the ACC also join the EmC in the human brain, adding an important criterion for segmenting this bundle. The EmC is the major pathway for the insula and superior temporal regions to connect with the rest of the cortex. Identifying the precise fiber composition in the EmC, including the regions they connect with, their spatial topography within the bundle, and their trajectory traversing the WM, is essential for understanding the EmC-connected functional networks and related brain disorders. Adding ACC fibers to the EmC calls for a re-consideration of its segmentation in future work.

### 4.3 SNR measurements for fODF reweighting

The effectiveness of fODF reweighting in this study suggests that SNR measurements can be informative for resolving complex fiber compositions when fitting the fODF. The SNR measurements are likely region sensitive, because different WM regions have different complications in their microstructure. In this work, we faced the complication that A-P–oriented thick bundles bias the SNR in a frontal WM region, while in some other regions such as the corpus callosum, this would not be the problem. A natural extension of the current study is to survey more WM regions and identify region-specific problems. For example, a voxel-wise SNR map of the entire WM can be informative. Moreover, the method to estimate SNR can be further developed. We used cross-subject variability as an SNR indicator by taking advantage of the large sample size of the HCP data. Potentially the fODF variability among neighboring voxels can achieve similar goals if there is a fiber passing each voxel in the same direction. Given this reasoning, the registration error across subjects is not a fundamental concern because nearby voxels contain similar information of the same underlying fibers. With voxels as samples, the SNR estimation may achieve greater statistical power, and machine-learning–related methods can be employed (e.g. Fadnavis et al., 2020). Finally, we note that, despite the many methods to separate signal from noise during data analysis, optimizing the acquisition parameters such as using higher b values would help boost the SNR and resolve crossing-fiber issues in general.

Another implication is that various fitting methods, such as those based on spherical deconvolution vs. biophysical models, will be affected differently by the underlying diffusivity asymmetry. The spherical deconvolution-based methods assume that the response function is rotationally symmetric and shared across all fiber populations. In this study, the reweighting is a post hoc modification to the spherical deconvolution results. For biophysical models that can accommodate asymmetric diffusivities, it is possible to incorporate a prior during fitting. For example, recent approaches that model fiber orientation distributions hierarchically (Rafipoor et al., 2025), using across-subject voxel-wise variability as a regularization prior, are well aligned with the cross-subject variance reweighting in the current study.

Finally, registration accuracy will be an important factor for estimating cross-subject variance. As mentioned above, in a relatively large deep WM region, we expect similarity of diffusivity orientations among neighboring voxels. However, this smoothness assumption may not hold for regions with heterogenous diffusivity properties (e.g., regions at the gray–white boundary). In such regions, accurate alignment across subjects would be critical for the reweighting approach to be effective. A multimodal registration approach (e.g., including FOD-based metrics or diffusion tensor alignment) may be more robust and particularly helpful.

### 4.4 Probabilistic vs. deterministic tractography for mapping the ACC–vlPFC/insula pathways

When mapping the ACC–vlPFC/insula pathways, probabilistic and deterministic approaches affect the results differently. Probabilistic approaches generally produce fewer false-negative but more false-positive results in crossing-fiber regions than deterministic approaches. This makes the probabilistic approach more appropriate for the current study, which aims to fix a false-negative problem by recovering M-L–oriented fibers that cross A-P–oriented bundles. Indeed, we observed proportionally more ACC–vlPFC connections with the probabilistic approach (Figs. 3A & 5A), despite the overall similar streamline patterns between the two approaches. However, the deterministic approach produced more streamlines in the IFOF than the probabilistic approach did. This was because the IFOF passes through the narrow WM region between the striatum and the insula (i.e., the region that overlaps with the EmC). In this region, because deterministic tractography follows the direction of the greatest diffusivity that is tangential to the GM surfaces, the resulting streamlines follow through the IFOF relatively easily. In contrast, probabilistic tractography generates samples in different directions, and the resulting streamlines often deviate from the tangential direction and terminate in the nearby GM. Put together, the deterministic approach is more helpful for inspecting streamline trajectories, while the probabilistic approach is more appropriate for calculating streamline density in the target regions. The two approaches complement each other in solving the ACC–vlPFC/insula tractography problem.

### 4.5 Cross-species analysis for improving human tractography

NHP tractography plays a critical role in linking human *in vivo* neuroanatomy with the NHP *ex vivo* ground truth from the literature. On one hand, NHP tractography results can be directly compared with tract-tracing findings (Girard et al., 2020). Such comparison validates the efficacy of dMRI tractography methods in the same species. On the other hand, NHP tractography can be compared with human tractography to provide additional information that is otherwise inaccessible in human *in vivo* studies. In previous work, this strategy has been applied to elucidate topographical rules, fingerprints, or network organization of anatomical connectivity in the human (Jbabdi et al., 2013; Neubert et al., 2015; Safadi et al., 2018; Tang et al., 2019). Here we further demonstrate that this cross-species, multimodal approach is effective in delineating detailed trajectories of individual bundles.

Finally, we would like to take a cautionary note on assessing technical issues against cross-species differences. Although we argue that, in this study, the low streamline density of the M-L connections and the absence of the entire EmC in the human brain reflect an amendable technical issue rather than a true anatomical difference across species, we do not rule out possible cross-species differences in the connectivity pattern of these pathways. For example, compared with the monkey ACC, the human ACC contains a substantially expanded cytoarchitectonic area 32. This difference may require the above-mentioned pathways to take species-specific trajectories to reach their target subregions in the ACC. Similar arguments apply to possible cross-species differences in the insula and vlPFC as well. Therefore, we note that there may not be a universal rule to separate cross-species differences caused by technical factors from those by anatomical dishomology, and that a good practice is to conduct such analyses on a case-by-case basis.

## Supplementary Material

Supplementary Material

## Data Availability

All data used are available at public databases as detailed in Section 2. Custom scripts used for fODF reweighting are available upon request to the corresponding author.
