# Supplementary material for: Mapping the structural connections between the anterior cingulate cortex and the insula/ventrolateral prefrontal cortex
Source: Imaging Neurosci (Camb). 2026 May 26;4:IMAG.a.1253. doi: 10.1162/IMAG.a.1253 (PMC13214568; doi:10.1162/IMAG.a.1253)
Supplement: Supplementary Material [file IMAG.a.1253_supp.pdf]

Supplemental Figures

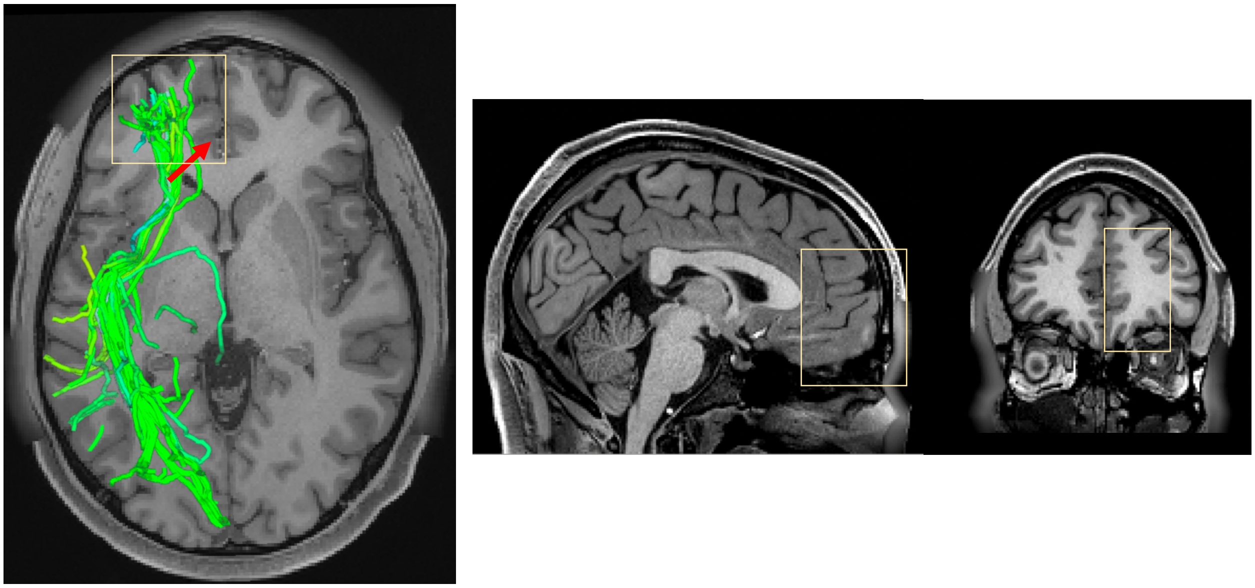

Supplemental figure 1. Streamlines in the IFOF from an extended seed region. Yellow rectangles mark the seed region. All the streamlines from this seed that pass through the IFOF are displayed with an axial slice. Red arrow marks the location of the original ACC seed.

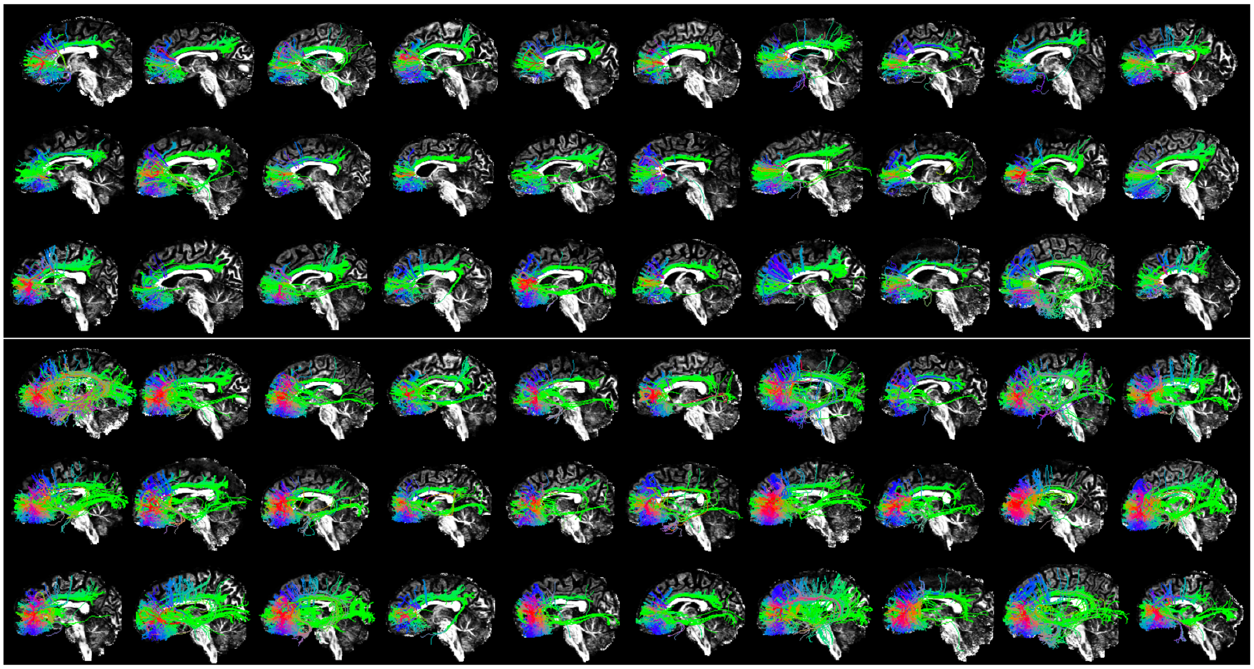

Supplemental figure 2. Tractograms of HCP subjects # 1-30. White line separates the results before (above line) and after (below line) fODF reweighting, with identical subject order.

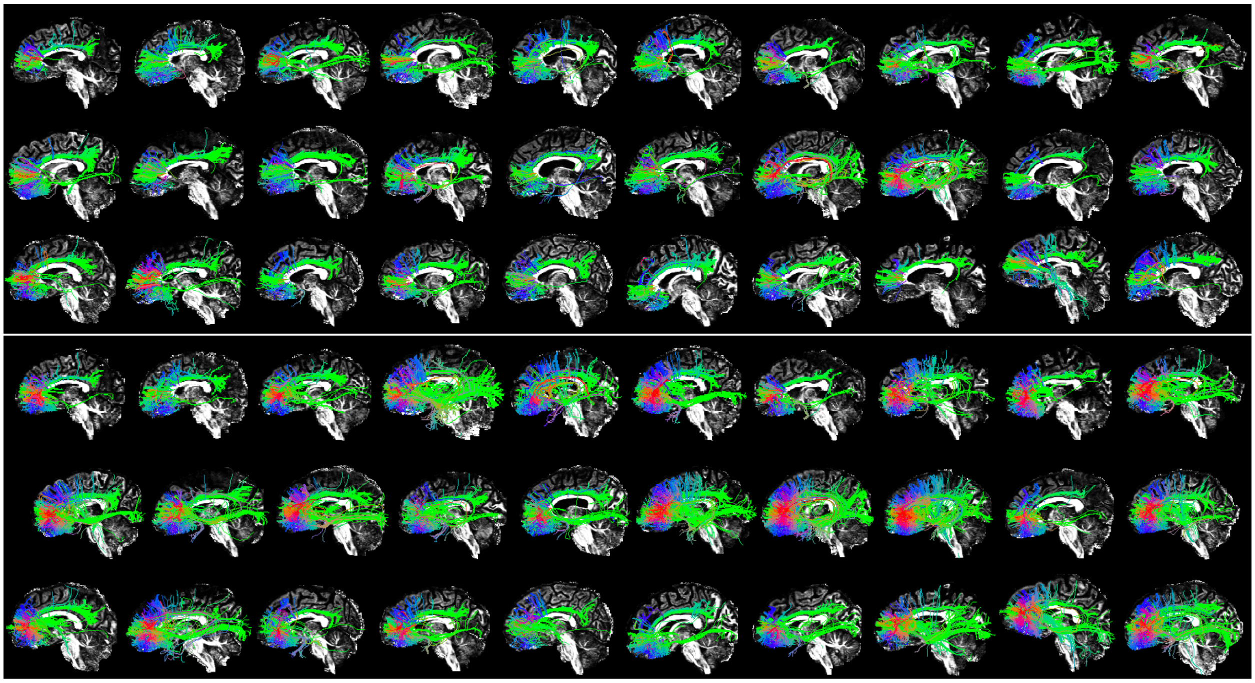

Supplemental figure 3. Tractograms of HCP subjects # 31-60. White line separates the results before (above line) and after (below line) fODF reweighting, with identical subject order.

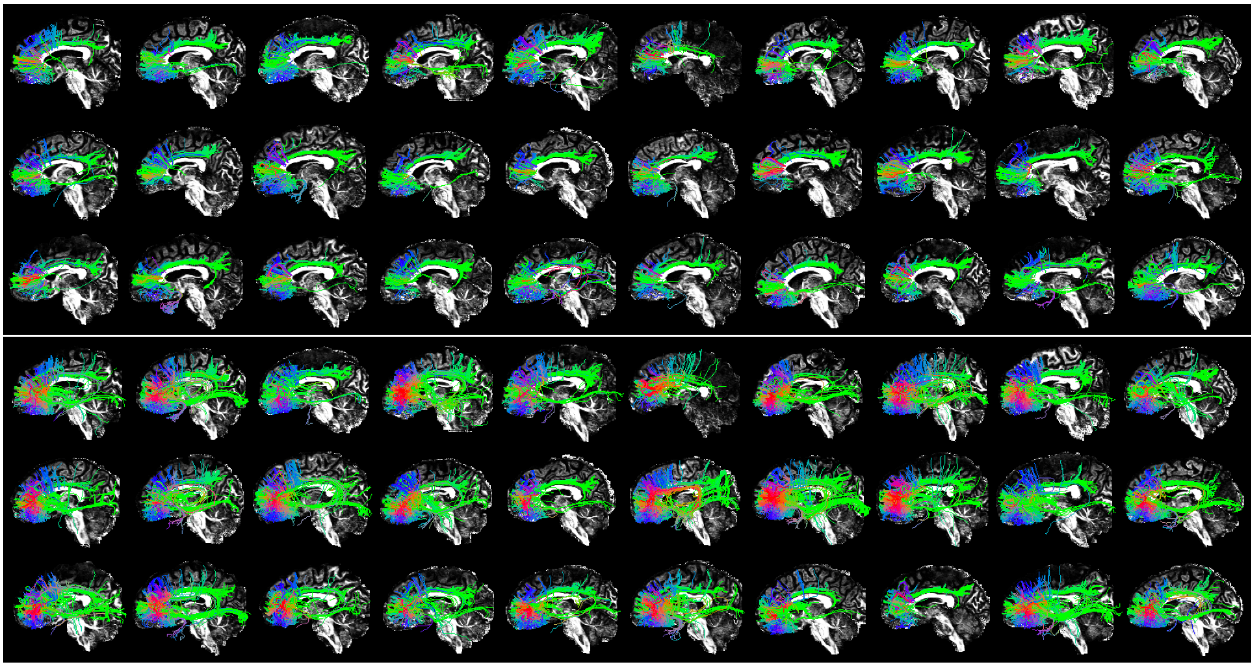

Supplemental figure 4. Tractograms of HCP subjects # 61-90. White line separates the results before (above line) and after (below line) fODF reweighting, with identical subject order.

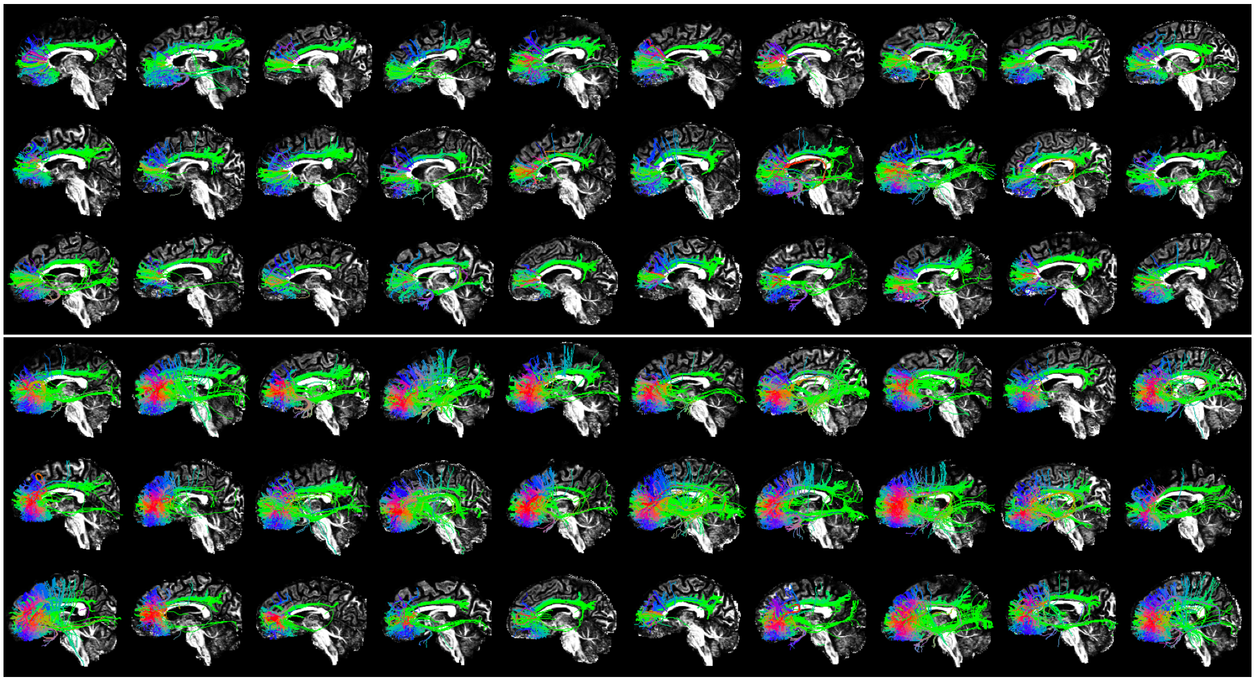

Supplemental figure 5. Tractograms of HCP subjects # 91-120. White line separates the results before (above line) and after (below line) fODF reweighting, with identical subject order.

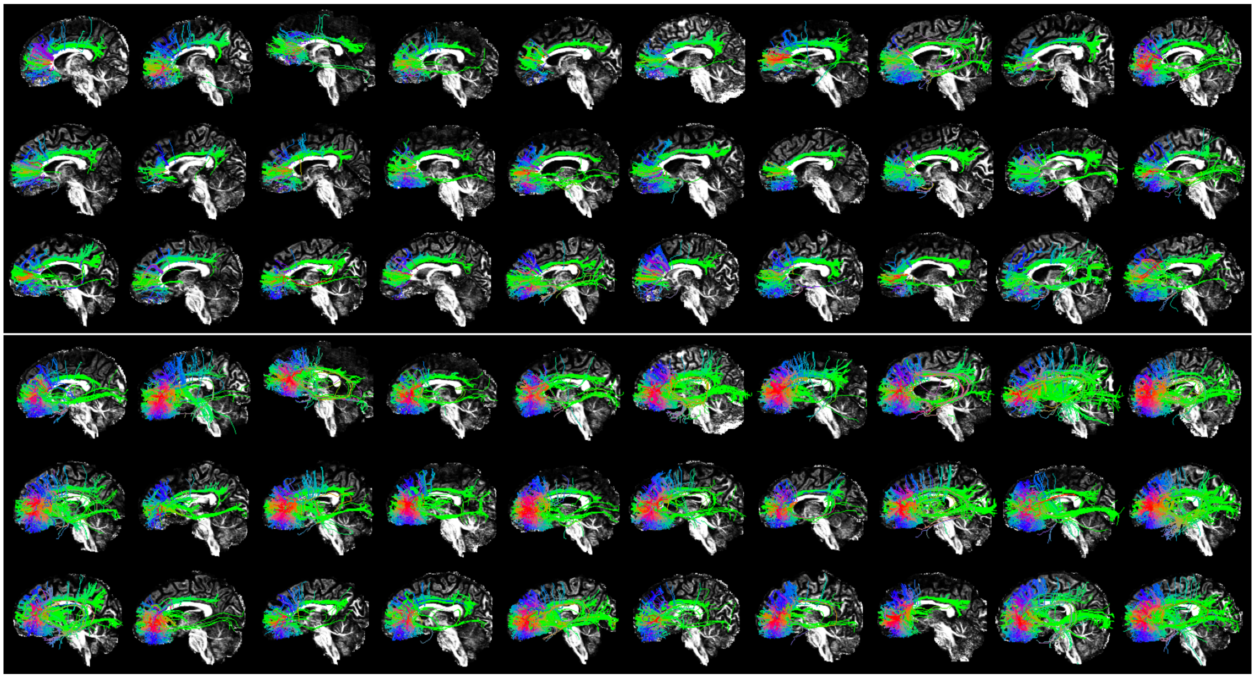

Supplemental figure 6. Tractograms of HCP subjects # 121-150. White line separates the results before (above line) and after (below line) fODF reweighting, with identical subject order.

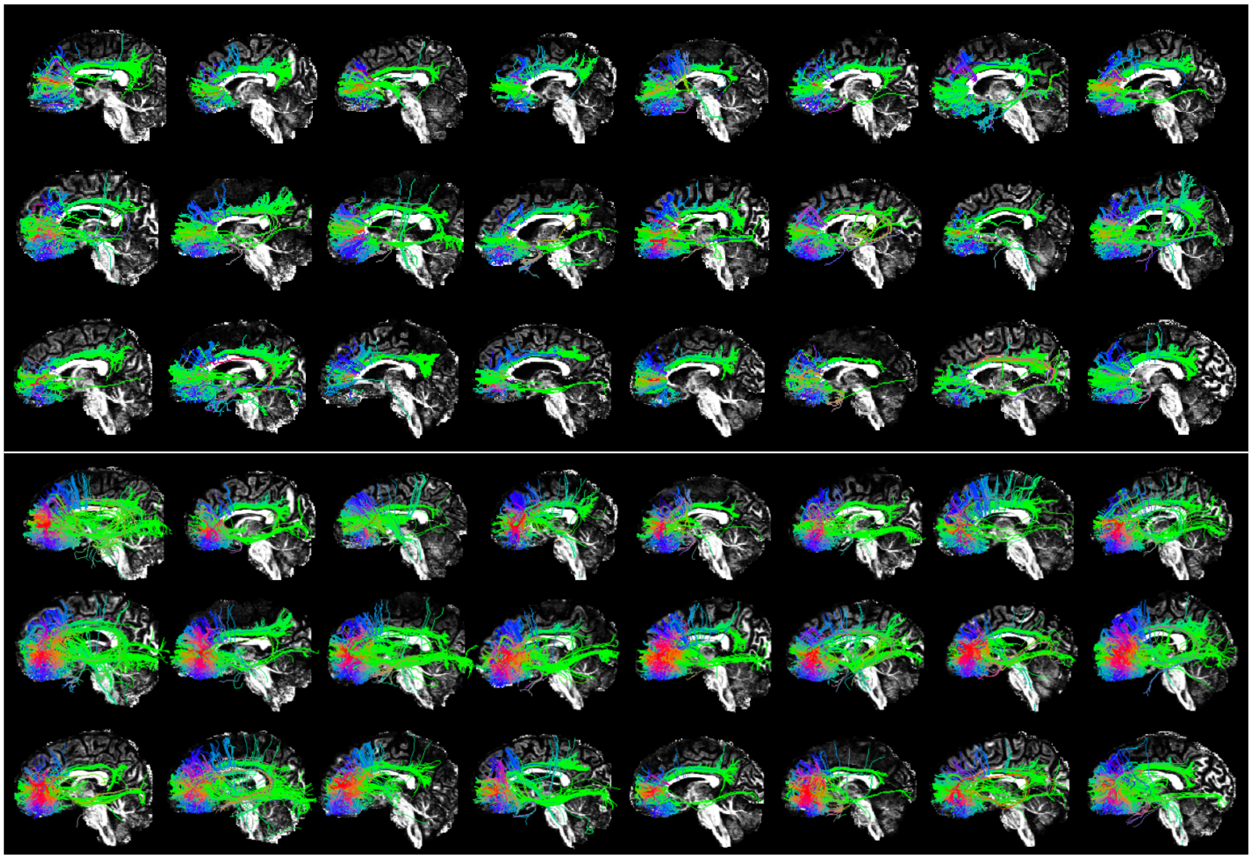

Supplemental figure 7. Tractograms of HCP subjects # 151-174. White line separates the results before (above line) and after (below line) fODF reweighting, with identical subject order.
